# Molecular alterations in key-regulator genes among patients with T4 breast carcinoma

**DOI:** 10.1186/1471-2407-10-458

**Published:** 2010-08-24

**Authors:** Bruno Massidda, MariaCristina Sini, Mario Budroni, Francesco Atzori, MariaCristina Deidda, Valeria Pusceddu, MariaTeresa Perra, Paola Sirigu, Antonio Cossu, Grazia Palomba, MariaTeresa Ionta, Giuseppe Palmieri

**Affiliations:** 1 Department of Medical Oncology, University of Cagliari, Cagliari, Italy; 2 Institute of Biomolecular Chemistry-National Research Council (CNR), Sassari, Italy; 3 Epidemiology Unit, Azienda Sanitaria Locale 1, Sassari, Italy; 4 Department of Cytomorphology, Cagliari University, Cagliari, Italy; 5 Institute of Pathology, Azienda Ospedaliero Universitaria, Sassari, Italy

## Abstract

**Background:**

Prognostic factors in patients who are diagnosed with T4 breast carcinomas are widely awaited. We here evaluated the clinical role of some molecular alterations involved in tumorigenesis in a well-characterized cohort of T4 breast cancer patients with a long follow-up period.

**Methods:**

A consecutive series of 53 patients with T4 breast carcinoma was enrolled between 1992 and 2001 in Sardinia, and observed up for a median of 125 months. Archival paraffin-embedded tissue sections were used for immunohistochemistry (IHC) and fluorescence *in situ *hybridization (FISH) analyses, in order to assess alterations in expression levels of survivin, p53, and pERK_1-2 _proteins as well as in amplification of *CyclinD1 *and *h-prune *genes. The Kaplan-Meier and Cox regression methods were used for survival assessment and statistical analysis.

**Results:**

Overall, patients carrying increased expression of pERK_1-2 _(p = 0.027) and survivin (p = 0.008) proteins as well as amplification of *h-prune *gene (p = 0.045) presented a statistically-significant poorer overall survival in comparison with cases found negative for such alterations. After multivariate analysis, the pathological response to primary chemotherapy and the survivin overexpression in primary carcinoma represented the main parameters with a role as independent prognostic factors in our series.

**Conclusions:**

Although retrospective, our study identified some molecular parameters with a significant impact on prediction of the response to therapy or prognosis among T4 breast cancer patients. Further large prospective studies are needed in order to validate the use of such markers for the management of these patients.

## Background

Since the staging systems of breast cancer were introduced during the course of the last century, the involvement of the skin has always been considered a morphologic characteristic leading to the classification of the tumour into the highest non-metastatic disease stage. In the current edition of the International Union Against Cancer (UICC)/American Joint Committee on Cancer (AJCC) TNM staging system [1], primary breast cancers with extension to the skin are classified as T4. Patients with T4 carcinomas of any type, with or without lymph node involvement, and without distant metastases (T4 N0-2 M0), are classified as disease stage IIIB. According to this system, the breast carcinoma with skin involvement is included in stage III and may be considered as locally-advanced breast cancer (LABC) [1-3].

In addition to the tumour size and the axillary lymph node involvement, other well-established prognostic factors currently used in breast cancer include histological subtype or grade, estrogen (ER) and progesterone (PR) receptor status, *HER2 *amplification, and Ki67 proliferation index [4,5]. Novel tumour markers with potential clinical utility are thus awaited.

The molecular mechanisms underlying locally-advanced breast carcinomas are largely unknown. A distinct gene-expression profile has been described for T3/T4 tumours in comparison to the gene-expression pattern of T1/T2 tumours [6], suggesting that a distinct biological behaviour may characterize initial *vs*. locally-advanced breast carcinomas. The mitogen activated protein kinase (MAPK) pathway, a major signalling cascade involved in the control of cell growth and proliferation, has been indicated to play a role in the intracellular signalling process of breast carcinomas [7-9]. The ERK1-2 proteins, which represent the final components of such a signalling kinase cascade, have been found to be activated through phosphorilation (pERK_1-2_) in human cancer and implicated in rapid malignant cell growth, mostly as a consequence of mutations in upstream components of the pathway [10,11]. Presence of pERK_1-2 _could be thus considered as a marker for the increased activity of ERK1-2, which may induce cell proliferation, rapid cancer cell growth, and resistance to apoptosis [10]. Moreover, a genomic instability with an increased number of copies of the *CyclinD1 *gene, which encodes a component of the p16^CDKN2A^-RB pathway functionally interacting with the MAPK pathway [12,13], has been described to promote a deregulation of the cell cycle with subsequent induction of an uncontrolled cell proliferation and tumour growth [14]. Nevertheless, the p53 protein represent the final effector of the p14^CDKN2A^-MDM2 pathway; in majority of human cancers, the *TP53 *gene is functionally inactivated [15]. Lack or reduced expression levels of the p53 protein seems to be associated with a defective apoptotic response to genotoxic damage and, thus, to anticancer agents [16].

Finally, two additional mechanisms seem to play a central role in breast cancer progression and resistance to treatment. The increased expression of survivin, a member of the inhibitor-of-apoptosis (IAP) protein family, has been demonstrated to be associated with resistance to apoptosis [17-19]. It has been reported that survivin and other IAP proteins cooperate to activate kinase cascades which control cell motility, thus stimulating tumour cell invasion and promoting metastasis [19]. Survivin is indeed overexpressed in most cancer cells and tissues of different histological origin, being correlated to overall survival and acting as a poor prognostic factor in some cancer patients [20-22]. In breast carcinomas, the up-regulation of survivin has been hypothesized to act as a factor exerting resistance against tamoxifen-induced apoptosis [23,24]. The second additional mechanism involved in breast cancer pathogenesis includes an increased activity of the human homologue of the *Drosophila *prune (h-prune), which belongs to a superfamily of phosphoesterases [25]. It has been demonstrated that h-prune is able to promote cell motility through either induction of its phosphodiesterase activity (very recently, a multi-domain adaptor protein, ASAP1, has been reported to stimulate the h-prune phosphodiesterase activity [26]) or interaction with specific protein partners (mainly, nm23-H1) [27-29]. The h-prune protein has been found expressed at higher levels in breast, colorectal, and gastric carcinomas, participating to the promotion of both tumour invasiveness and metastasis formation [25,27]. In breast cancer, overexpression of h-prune has been demonstrated to be involved in cancer progression, identifying subsets of patients with higher tumour aggressiveness (although it seems to have no role as independent prognostic factor in clinical outcome of patients with invasive breast carcinoma) [30]. As previously demonstrated [25,30], gene amplification may play an important role in inducing overexpression of h-prune among breast cancer patients.

In the present study, we examined the expression of survivin, p53, and pERK_1-2 _proteins as well as the amplification of *CyclinD1 *and *h-prune *genes in a well-characterized cohorts of patients with T4 breast carcinoma and a long follow-up, in order to determine their association with clinical and pathological parameters as well as with patients' outcome.

## Methods

### Cases and tissue samples

Paraffin-embedded samples of 53 consecutive patients with T4 breast cancer were included into the study. Cases were enrolled between 1992 and 2001, and observed up to September 2008 for a median of 125 months (range, 82-194). Patients were assessed by physical examination and mammography, confirmed via core-needle biopsy. All patients completed a treatment plan including primary chemotherapy, surgery, radiation therapy, adjuvant chemotherapy, and hormone therapy, when indicated (see below). The median age was 51 years (range, 32-67). Baseline characteristics are summarized in Table 1.

**Table 1:** Patient and tumour characteristics at baseline

*Characteristics*	*Patients*	
	N	%
**Age**		
<50	**23**	43
>50	**30**	57
**Tumor stage**		
T4abc	**38**	72
T4d	**15**	28
**Axillary lymph nodes **		
N0	**0**	0
N+	**53**	100
**Hormone receptor status**		
ER+/ER-	**28/25**	53/47
PR+/PR-	**17/36**	32/68
**Proliferative index**		
Ki67+	**17**	32
Ki67-	**27**	51
unknown	**9**	17
**Tumour Grading**		
G2/G3	**38/15**	72/28
**HER2 status**		
HER2+	**10**	19
HER2-	**43**	81

Fifteen patients (28%) had initially inflammatory breast carcinoma (T4d) and 38 (72%) had initially non-inflammatory cancer (T4abc); all patients had clinical involvement of axillary limph nodes (as N+). According to the American Joint Committee on Cancer (AJCC) TNM staging system [1], all 53 cases included into this study were classified with the highest stage of non-metastatic disease (Stage IIIB). Estrogen (ER) and progesterone (PR) status was assessed by standard immunohistochemistry; nuclear staining in ≥10% was considered positive (according to the indication that a significant difference in 5-year recurrence-free survival between ER-positive and ER-negative patients has been reported for a cut-off of 10% [31]). HER2 status was assessed by fluorescence *in situ *hybridization (FISH) analysis.

The study was approved by the Institutional Review Board at the University of Cagliari. A written informed consent was obtained for using tissue specimens in molecular analyses.

### Treatment plan

All patients were treated with primary chemotherapy using anthracyline-containing regimens, such as FEC (5-Fluorouracil; Epirubicin; Cyclophosphamide) or PEV (Cisplatin; Epirubicin; Vinorelbine). After completing the neoadjuvant chemotherapy, patients underwent surgery consisting of modified radical mastectomy (MRM) or breast-conserving surgery (BCT). Postoperative adjuvant chemotherapy consisted of six cycles of CMF (cyclophosphamide, methotrexate, fluorouracil). Locoregional radiotherapy was performed during the fourth course of CMF. After completing adjuvant chemotherapy, patients with hormone receptor-positive tumours received tamoxifen for 5 years.

Clinical evaluations were performed every 3 months for 2 years and every 6 months thereafter. Instrumental examinations (e.g., mammography, liver ultrasound, chest X-ray, bone scan, and echocardiogram) were performed every 6 months for the first 2 years, and every 12 months thereafter.

### Response Assessment

The clinical measurement of the response to neoadjuvant therapy was defined according to the International Union Against Cancer (UICC) criteria [32]. Pathological complete response (pCR) was defined as the histological absence of residual invasive disease in both the breast and the axilla. Presence of histological invasive residual disease in breast tissue or detection of cancer-positive lymph nodes in the axilla were defined as <pCR. Major pathological response (MpR) in breast tissue was defined as no more than 2 cm of residual disease (pT0 plus pT1) [27].

### Immunohistochemistry (IHC)

Immunohistochemical staining was done on formalin-fixed, paraffin-embedded sections, as previously described [30]. Four- to five-micrometer sections were immunostained with each specific monoclonal antibody (anti-survivin, anti-p53, and anti-phosphorilated ERK_1-2_). Slides were viewed using a BX61 Olympus Microscope supplied with DP50 camera and Viewfinder Lite 1.0 Version (Pixera Corporation) image analysis system. Labelling intensity and cellular staining was independently evaluated by two observers. Intensity and distribution of IHC staining was used to classify samples as positive (tissue sections presenting strong to moderate staining in more than 10% of cells) or negative (including tissue sections showing weak to absent staining) for expression of candidate genes.

### Fluorescence in situ Hybridization (FISH)

For *h-prune *and *CyclinD1 *gene amplification analysis, double-colour FISH analysis was performed using the PAC 279-H19 clone, spanning the *h-prune *gene region at chromosome 1q21, and the BAC RP11-300I6 clone specific for the *CyclinD1 *gene at chromosome 11q13, according to previously reported protocols [25,30]. Nuclei were counterstained with 4',6-diamidino-2-phenyl-indole (DAPI). Three distinct experiments were performed for each case. Digital images were captured using an Olympus BX-61 epifluorescence microscope, equipped with the appropriate filters, a COHU video, and the Cytovision software.

Hybridization signals on at least 100 intact, well-preserved, and non-overlapping nuclei were evaluated by at least two investigators. A gain of gene copy was defined as presence of multiple (three or more) signals in at least 10% of nuclei

### Statistical analysis

Chi-square and Fisher's exact tests were used to evaluate possible associations between covariates (ER; PR; Ki67 proliferative index; HER2; expression for survivin, p53, and phosphorilated ERK_1-2_; amplification of h-prune and Cyclind1) and clinical outcome in terms of treatment responses and median survivals. Univariate correlations between prognostic variables and survival outcomes were carried out using the Kaplan-Meier method. Variables were also evaluated for independent correlations on survival by Cox regression analysis. Statistical comparisons were performed using the SPSS statistical software package, version 15.0 (SPSS Inc., Chicago, IL, USA). All tests were two-tailed and P values of less than 0.05 were considered to be statistically significant.

## Results

### Patients' collection

To evaluate the pathogenetic and prognostic roles of five candidate molecular markers (expression levels of survivin, p53, and pERK1-2 proteins; amplification of *CyclinD1 *and *h-prune *genes), we have examined fifty-three patients with diagnosis of T4 breast carcinoma (T4-N0-2-M0, according to the TNM classification by Sobin *et al. *[1]).

All patients were evaluated for response to primary chemotherapy. No disease progression was observed during the treatment. The clinical response rate was 100% (95% CI, 65,2-89,5): a complete clinical response was observed in 8 patients (15%). According to Sataloff's classification [33], pathological complete response in primary tumour (pCR) was observed in 8 patients (15%); major pathological response (MpR), corresponding to pT0-pT1 classification after primary chemotherapy, was observed in 18 (34%) breast tissues. The pathological lymph node assessment revealed absence of involvement (pN0) in 12 (23%) patients after primary chemotherapy.

All 53 patients became suitable for surgery. Modified radical mastectomy was performed in 36 patients (68%) and breast conserving treatment (BCT) was feasible in 17 patients (32%). Records of the clinical follow-up covered a median period of 125 months (range 70-182); 10-year disease-free survival (DFS) and overall survival (OS) within the entire series were 32% and 43%, respectively.

### Immunohistochemistry and FISH analysis

Assessment of the expression levels of survivin, p53, and pERK_1-2 _proteins by immunohistochemistry (IHC) as well as characterization of the chromosomal copy number of *cyclinD1 *and *h-prune *genes by fluorescence *in situ *hybridization (FISH) analysis was carried out on paraffin-embedded tissue sections from primary breast carcinomas of our series.

**Figure 1:**
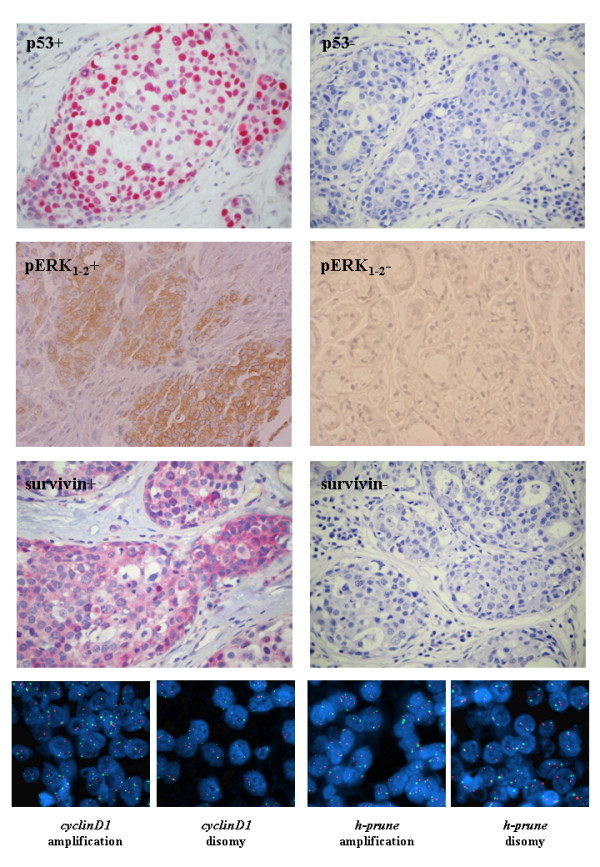
**Immunohistochemistry and FISH analysis**. (Up-middle) Typical examples of T4 breast carcinoma tissue sections positive (left) or negative (right) for p53, pERK_1-2_, and survivin protein expression. (Bottom) Typical examples of double-colour FISH results. Nuclei extracted from paraffin-embedded tissues after hybridization with probes specific for *cyclinD1 *or *h-prune *loci (red signals) and control chromosome centromeres (green signals).

For IHC analysis, a lack of expression for the p53 protein was observed in 13/53 (25%) patients; conversely, a positive immunostaining was detected in 5/53 (9%) cases for pERK_1-2_, and 21/53 (40%) cases for survivin (Table 2). The FISH analysis was performed using specific probes corresponding to the *h-prune *and *cyclinD1 *genomic regions at chromosomes 1q21 and 11q13 (respectively) as well as control clones spanning the peri-centromeric regions at chromosomes 1 and 11, respectively. Multiple FISH signals in >10% analyzed nuclei were found in 8/53 (15%) cases, for *h-prune*, and 12/53 (23%) cases, for *cyclinD1 *(Table 2). A normal copy number (diploid signals) was detected for centromeric control probes, confirming the specificity of the amplification at 1q21 and 11q13 loci and excluding any procedure artifact. Absence of karyotypic anomalies in cells from normal tissues surrounding the tumours strongly indicated that amplification of the *h-prune *and *cyclinD1 *genomic regions was highly specific for breast cancer cells. Representative examples of IHC staining and FISH results are shown in Figure 1.

**Table 2:** Comparison between IHC or FISH results and histopathological parameters

Characteristics	*FISH analysis*				*IHC analysis*					
	*CyclinD1*		*h-prune*		p53		pERK_1-2_		survivin	
	positive cases	%	positive cases	%	positive cases	%	positive cases	%	positive cases	%
**Total patients **(N = 53)	**12**	23	**8**	15	**13**	25	**5**	9	**21**	40

**Estrogen receptor (ER)**										
negative (N = 20)	**2**	10	**1**	5	**4**	20	**2**	10	**9**	45
positive (N = 24)	**7**	29	**5**	11	**5**	21	**2**	8	**7**	29

**Progesterone receptor (PR)**										
negative (N = 28)	**5**	18	**2**	7	**5**	18	**2**	7	**13**	46
positive (N = 16)	**4**	25	**4**	25	**4**	25	**2**	12	**3**	19

**Ki67**										
negative (N = 26)	**4**	15	**3**	12	**4**	15	**4**	15	**11**	42
positive (N = 16)	**4**	25	**2**	12	**5**	31	**0**	0	**5**	31

**HER2**										
0-1 (N = 16)	**5**	31	**3**	19	**2**	12	**2**	12	**9**	56
2-3 (N = 37)	**7**	19	**5**	14	**11**	30	**3**	8	**12**	32

### Correlation with clinico-pathological parameters

Using Pearson's Chi-Squared test, molecular alterations were evaluated for association with histological tumour characteristics: ER and PR status, *HER2 *amplification, Ki67 proliferation index. No statistically significant correlation between any of the molecular alteration and pathological parameters was observed (including triple negative tumours: ER-, PR-, HER2-), with the exception of the detection of positive pERK_1-2 _immunostaining in the group of patients negative for Ki67 expression only [the pERK_1-2 _expression was not detected in tumours expressing Ki67 (0/16), whereas 4/26 (15%) Ki67+ tumours presented a positive pERK_1-2 _immunostaining; p = 0.041] (Table 2).

To investigate the role in predicting the response to primary chemotherapy, all tumour characteristics (histological parameters and molecular alterations) were compared to clinical and pathological outcome in our series. As shown in Table 3, the Ki67 proliferation index and *HER2 *amplification were significantly associated with a better clinical outcome [5/7 (71%) complete clinical responses *vs*. 11/35 (31%) partial clinical responses, for Ki67 proliferation index; 8/8 (100%) complete clinical responses *vs*. 29/45 (64%) partial clinical responses, for *HER2 *amplification]. Conversely, the expression of pERK_1-2 _was significantly associated with a worse clinical outcome [0/8 complete clinical response *vs*. 5/45 (11%) partial clinical responses] (Table 3). Considering the histological classification, a higher prevalence of positive Ki67 proliferation index was found in cases achieving the pathological response [5/7 (71%) pCR *vs*. 11/35 (31%) non-pCR]; a positive pERK_1-2 _expression was instead observed in patients who did not achieve the pathological response [0/8 pCR *vs*. 5/45 (11%) non-pCR] (Table 3).

**Table 3:** Comparison between histopathological or molecular parameters and response to therapy

A															
***Outcome***	***ER***			***PR***			***Ki67***			***HER2***					
	**positives**	**%**	***P***	**positives**	**%**	***P***	**positives**	**%**	***P***	**positives**	**%**	***P***			

**Clinical response**			*0.132*			*0.640*			*0.047*			*0.022*			
Complete response	**3/7**	43		**3/7**	43		**5/7**	71		**8/8**	100				
Partial response	**16/29**	55		**11/29**	38		**9/27**	33		**27/37**	73				
< Partial response	**5/8**	62		**2/8**	25		**2/8**	25		**2/8**	25				

**Pathological response**			*0.469*			*0.235*			*0.042*			*0.095*			
pCR	**4/7**	57		**3/7**	43		**5/7**	71		**7/8**	87				
<pCR	**20/37**	54		**13/37**	35		**11/35**	31		**30/45**	67				

**B**															

***Outcome***	***cyclinD1***			***h-prune***			**pERK_1-2_**			**p53**			**survivin**		
	**positives**	**%**	***P***	**positives**	**%**	***P***	**positives**	**%**	***P***	**positives**	**%**	***P***			

**Clinical response**			*0.457*			*0.824*			*0.032*			*0.391*			*0.089*
Complete response	**1/8**	12		**1/8**	12		**0/8**	0		**1/8**	12		**1/8**	12	
Partial response	**8/37**	22		**5/37**	14		**3/37**	8		**10/37**	27		**15/37**	41	
< Partial response	**3/8**	37		**2/8**	25		**2/8**	25		**2/8**	25		**5/8**	62	

**Pathological response**			*0.660*			*0.822*			*0.007*			*0.972*			*0.234*
pCR	**2/8**	25		**1/8**	12		**0/8**	0		**2/8**	25		**3/8**	37	
<pCR	**10/45**	22		**7/45**	16		**5/45**	11		**11/45**	24		**18/45**	40	

pCR, pathological complete response

**Figure 2:**
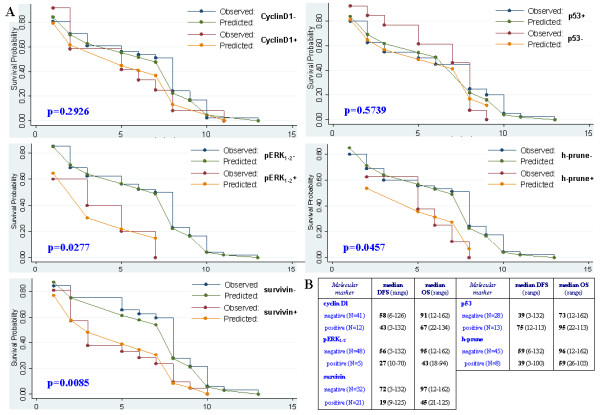
**Comparison between molecular markers and survivals among T4 breast cancer patients**. (A) Overall survival curves based on the Kaplan-Meier method. Statistical comparison between observed and predicted survival data for each subset of patients [with (+) or without (-) alterations in expression of p53, pERK_1-2 _and survivin proteins or in copy number of *h-prune *and *cyclinD1 *genes) is reported. (B) Median disease-free and overall survivals (DFS and OS, respectively), expressed in months, for each subset of patients.

Each molecular alteration was then evaluated for its impact on overall survival. Using the Kaplan-Meier method, survival curves indicated that patients carrying pERK_1-2 _positive staining (p = 0.027), *h-prune *amplification (p = 0.045), and survivin overexpression (p = 0.008) presented a statistically-significant poorer overall survival in comparison with those resulted negative for such alterations (Figure 2A). No significant association with overall survival was observed for p53 down-expression and *cyclinD1 *amplification (Figure 2A). As summarized in Figure 2B, median overall survivals were consistently higher in breast cancer patients with absence of *h-prune *amplification (median OS: 96 months in comparison to 59 months of patients with *h-prune *polysomy) and negative immunostaining for pERK_1-2 _(median OS: 95 months in comparison to 43 months of patients with pERK1-2+ tumours) and survivin (median OS: 97 months in comparison to 45 months of patients with survivin overexpression).

Using the Cox model adjusted according to age at diagnosis for a multivariate analysis, pathological response to primary chemotherapy and survivin overexpression remained the only parameters with a significant impact on prognosis in our series of breast cancer patients; no other association with overall survival was observed for the remaining variables (Table 4).

**Table 4:** Multivariate analysis of different parameters for overall survival

*Characteristic*	*Hazard Ratio*	*95% CI*	*P*
*cyclinD1*	0.75	0.26-2.12	0.591
*h-prune*	2.37	0.85-7.03	0.078
pERK_1-2_	1.08	0.37-3.18	0.875
p53	0.74	0.34-1.58	0.443
**survivin**	**3.40**	**1.20-4.76**	**0.012**
estrogen receptor (ER)	1.88	0.57-6.91	0.281
progesterone receptor (PR)	1.33	0.36-4.84	0.639
proliferation index (Ki67)	1.81	0.48-6.85	0.377
HER2	3.24	0.96-14.5	0.059
**pathological response**	**8.83**	**1.10-50.2**	**0.040**

Stratified by age. CI, confidence interval. In bold, significant values.

## Discussion

In this study, we evaluated the impact of some specific molecular alterations (activation of ERK1-2 proteins, amplification of *CyclinD1 *and *h-prune *genes, silencing of *TP53 *gene, overexpression of survivin protein) as predictive and prognostic factors among patients with T4 breast carcinoma. The analyzed molecular alterations have been largely demonstrated to play an important role in: *a*) deregulating the cell cycle with subsequent induction of abnormal cell proliferation and tumour growth (ERK1-2 phosphorilation and *CyclinD1 *amplification); *b*) impairing the apoptotic machinery with subsequent induction of resistance to anticancer agents (p53 downexpression and survivin overexpression); and *c*) promoting metastasis formation (*h-prune *amplification). Our findings indicated that subsets of T4 breast cancer patients with pERK_1-2 _staining, survivin expression, or *h-prune *amplification in primary tumour tissues presented a worse overall survival. After multivariate analysis, the pathological response to primary chemotherapy and the survivin overexpression in primary carcinoma represented the main parameters with a role as independent prognostic factors predicting the clinical outcome in such a series of breast cancer patients.

Although an increased expression of survivin in tumour tissues has been already demonstrated to correlate with a poor clinical outcome in a variety of malignancies [20-22], our results clearly indicated an analogous significant impact on prognosis of such a molecular alteration among T4 breast cancer patients. From the pathogenetic point of view, survivin has been found to provide protection against apoptotic stimuli by inhibiting activation of caspase-9 toward the initiation of the intrinsic mitochondrial pathway of apoptosis [18]. Recently, it has been demonstrated that survivin as well as other members of the IAP protein family are strongly involved in metastasis formation; search for survivin-IAP antagonists may indeed provide new antimetastatic therapies for cancer patients [19]. Nevertheless, survivin seems to be upregulated through the activation of the MAPK-ERK pathway [34]; in other words, the overexpression of survivin may be associated with the increased levels of ERK1-2 phosphorylation (in our series, all 5 cases expressing pERK_1-2 _proteins also presented survivin overexpression). Interestingly, our findings indicated that presence of pERK_1-2 _expression in primary T4 carcinomas may be indeed correlated with clinical outcome (see Figure 2), suggesting that the cascade of molecular events activating ERK1-2 and upregulating survivin has indeed an important prognostic role in such patients. One could speculate that the lack of a significant association with prognosis for pERK_1-2 _staining in multivariate analysis may be due to the fact that we identified only a limited fraction (5/53; 9%) of carriers and, thus, the subgroup analysis relied on a small number of subjects.

The well-established prognostic factors currently used into the management of breast cancer patients include the disease stage as well as the degree of differentiation (tumour grade), the proliferation index, and the hormone receptor status (ER, PR, and, recently, HER2) in primary tumours [35,36]. In our series of patients with T4 breast carcinoma, no statistically-significant correlation between any of the analyzed molecular alterations and such pathological parameters was inferred. The only exception was represented by the correlation between the pERK_1-2 _staining and the Ki67 proliferation index. None of the tumours expressing a high Ki67 proliferation index showed an increased level of pERK_1-2 _protein; conversely, all cases with activated ERK_1-2 _protein presented a low Ki67 proliferation index. Activation of ERK1-2 proteins has been demonstrated to promote cell cycle progression, participating to induction of cell growth and enhancement of cell survival [10]. Our findings led us to speculate that: a) induction of cell proliferation via pERK_1-2 _and Ki67 molecules may represent two unrelated phenomena; and b) among patients with low Ki67 expression levels (who may have an unfavourable prognosis [37], though the role of Ki67 proliferation index as prognostic and predictive marker is yet to be conclusively defined [38]), the presence of pERK_1-2 _overexpression seems to identify a subgroup with an even worse prognosis. Taking into consideration the response rates, patients whose tumours had high Ki67 expression levels or *HER2 *amplification presented the highest rates of response to primary chemotherapy (for Ki67, a significant association was found with both clinical and pathological responses; for *HER2*, a significant association was surprisingly observed with clinical response only) (see Table 3). These latter findings are consistent with data previously reported [39-41]. Among the molecular parameters, only pERK_1-2 _expression seemed to be significantly correlated with response to primary chemotherapy (significant lower rates were observed for both clinical and pathological responses; see Table 3), reflecting the fact that the activation of ERK1-2 proteins may increase the resistance to apoptosis, reducing the sensitivity to chemotherapy [10].

Several mechanisms have been recently described to participate in progression of breast cancer through activation of the h-prune complex. It is now clear the existence of a network of interacting proteins which indeed regulate the phosphodiesterase activity of h-prune, contributing to promote (ASAP1) or inhibit (nm23-H1) either cancer cell motility and tumour adhesiveness *in vitro *either tumour invasiveness and metastasis formation *in vivo *[25-29]. The increased expression of h-prune protein has been demonstrated to deeply modify this equilibrium of opposite stimuli, playing an important role in promotion of cancer progression [25]. Among others, the main mechanism leading to h-prune overexpression is represented by the amplification of gene copy number [25,42]. Considering tumours with at least three gene copies, a small fraction (8/53; 15%) of T4 breast carcinomas from our series presented *h-prune *amplification at chromosome 1q21.3 (see Table 2); such a frequency is quite identical to that described in our previous report (173/1,016; 17%) [30]. All breast cancer patients included into the present study showed axillary nodal involvement; among them, occurrence of *h-prune *amplification was able to identify a subset with a worse overall survival (see Figure 2). As for pERK_1-2 _staining, the low number of events could explain the absence of a significant association of the *h-prune *amplification with prognosis in the multivariate analysis.

## Conclusions

Although our study was retrospective, some important indications about either the prediction of the response to therapy or the role on prognosis in T4 breast cancer patients have been inferred. There is no doubt that the pathological response after primary chemotherapy remains one of the major predictor of survival; however, the molecular marker represented by survivin overexpression may be also considered as a useful prognostic factor in these patients. To validate the incorporation of survivin or the other promising molecular parameters (h-prune and pERK_1-2_) as markers for management of T4 patients, further large prospective studies are awaited. Nevertheless, translational studies investigating additional molecular biomarkers should contribute to more accurately identify subsets of patients who would be expected to be more or less likely to respond to specific therapeutic interventions.

## List of abbreviations

AJCC: American Joint Committee on Cancer; ER; Estrogen Receptor; FISH: fluorescence *in situ *hybridization; IHC: immunohistochemistry; MAPK: mitogen activated protein kinase; PR: Progesterone Receptor; UICC: International Union Against Cancer.

## Competing interests

The authors declare that they have no competing interests.

## Authors' contributions

BM conceived of the study. MS performed molecular analysis. MB performed statistical analysis. FA participated to collection of cases. MD participated to collection of cases. VP participated to collection of cases. MP performed molecular analysis. PS participated to interpretation of results. AC participated to data management. GrP performed some molecular analyses. MI participated to design of the study. GiP participated to interpretation of data and drafted the manuscript.

All authors read and approved the final manuscript.

## Pre-publication history

The pre-publication history for this paper can be accessed here:

http://www.biomedcentral.com/1471-2407/10/458/prepub

## References

[B1] Sobin L Wittekind C (eds) TNM classification of malignant tumors 2002 6 John Wiley & Sons, New York

[B2] Singletary SE Allred C Ashley P Bassett LW Berry D Bland KI Borgen PI Clark G Edge SB Hayes DF Hughes LL Hutter RV Morrow M Page DL Recht A Theriault RL Thor A Weaver DL Wieand HS Greene FL Revision of the American Joint Committee on Cancer staging system for breast cancer J Clin Oncol 2002 20 3628 36 10.1200/JCO.2002.02.026 12202663

[B3] Woodward WA Strom EA Tucker SL McNeese MD Perkins GH Schechter NR Singletary SE Theriault RL Hortobagyi GN Hunt KK Buchholz TA Changes in the 2003 American Joint Committee on Cancer staging for breast cancer dramatically affect stage-specific survival J Clin Oncol 2003 21 3244 8 10.1200/JCO.2003.03.052 12947058

[B4] Subramaniam DS Isaacs C Utilizing prognostic and predictive factors in breast cancer Curr Treat Options Oncol 2005 6 147 159 10.1007/s11864-005-0022-1 15717996

[B5] Hayes DF Prognostic and predictive factors revisited Breast 2005 14 493 499 10.1016/j.breast.2005.08.023 16239111

[B6] Van Laere S Van der Auwera I Van den Eynden G Van Hummelen P van Dam P Van Marck E Vermeulen PB Dirix L Distinct molecular phenotype of inflammatory breast cancer compared to non-inflammatory breast cancer using Affymetrix-based genome-wide gene-expression analysis Br J Cancer 2007 97 1165 1174 10.1038/sj.bjc.6603967 17848951 PMC2360452

[B7] Santen RJ Song RX McPherson R Kumar R Adam L Jeng MH Yue W The role of mitogen-activated protein (MAP) kinase in breast cancer J Steroid Biochem Mol Biol 2002 80 239 256 10.1016/S0960-0760(01)00189-3 11897507

[B8] Eralp Y Derin D Ozluk Y Yavuz E Guney N Saip P Muslumanoglu M Igci A Kücücük S Dincer M Aydiner A Topuz E MAPK overexpression is associated with anthracycline resistance and increased risk for recurrence in patients with triple-negative breast cancer Ann Oncol 2008 19 669 74 10.1093/annonc/mdm522 18006896

[B9] McGlynn LM Kirkegaard T Edwards J Tovey S Cameron D Twelves C Bartlett JM Cooke TG Ras/Raf-1/MAPK pathway mediates response to tamoxifen but not chemotherapy in breast cancer patients Clin Cancer Res 2009 15 1487 95 10.1158/1078-0432.CCR-07-4967 19228750

[B10] Davies H Bignell GR Cox C Stephens P Edkins S Clegg S Teague J Woffendin H Garnett MJ Bottomley W Davis N Dicks E Ewing R Floyd Y Gray K Hall S Hawes R Hughes J Kosmidou V Menzies A Mould C Parker A Stevens C Watt S Hooper S Wilson R Jayatilake H Gusterson BA Cooper C Shipley J Hargrave D Pritchard-Jones K Maitland N Chenevix-Trench G Riggins GJ Bigner DD Palmieri G Cossu A Flanagan A Nicholson A Ho J Leung SY Yuen ST Weber BL Seigler HF Darrow TL Paterson H Marais R Marshall CJ Wooster R Stratton MR Futreal PA Mutations of the BRAF gene in human cancer Nature 2002 417 949 954 10.1038/nature00766 12068308

[B11] McCubrey JA Steelman LS Abrams SL Lee JT Chang F Bertrand FE Navolanic PM Terrian DM Franklin RA D'Assoro AB Salisbury JL Mazzarino MC Stivala F Libra M Roles of the RAF/MEK/ERK and PI3K/PTEN/AKT pathways in malignant transformation and drug resistance Adv Enzyme Regul 2006 46 249 279 10.1016/j.advenzreg.2006.01.004 16854453

[B12] Deng Q Liao R Wu BL Sun P High intensity ras signaling induces premature senescence by activating p38 pathway in primary human fibroblasts J Biol Chem 2004 279 1050 1059 10.1074/jbc.M308644200 14593117

[B13] Wen-Sheng W ERK signaling pathway is involved in p15INK4b/p16INK4a expression and HepG2 growth inhibition triggered by TPA and Saikosaponin a Oncogene 2003 22 955 963 10.1038/sj.onc.1206237 12592382

[B14] Vogelstein B Kinzler KW Cancer genes and the pathways they control Nat Med 2004 10 789 799 10.1038/nm1087 15286780

[B15] Pomerantz J Schreiber-Agus N Lié geois NJ The Ink4a tumor suppressor gene product, 19Arf, interacts with MDM2 and neutralizes DM2's inhibition of p53 Cell 1998 92 713 723 10.1016/S0092-8674(00)81400-2 9529248

[B16] Levesque AA Eastman A p53-based cancer therapies: Is defective p53 the Achilles heel of the tumor? Carcinogenesis 2007 28 13 20 10.1093/carcin/bgl214 17088261

[B17] Li F Ambrosini G Chu EY Plescia J Tognin S Marchisio PC Altieri DC Control of apoptosis and mitotic spindle checkpoint by survivin Nature 1998 396 580 10.1038/25141 9859993

[B18] Dohi T Beltrami E Wall NR Plescia J Altieri DC Mitochondrial survivin inhibits apoptosis and promotes tumorigenesis J Clin Invest 2004 114 1117 1127 15489959 10.1172/JCI22222 PMC522254

[B19] Mehrotra S Languino LR Raskett CM Mercurio AM Dohi T Altieri DC IAP regulation of metastasis Cancer Cell 2010 17 53 64 10.1016/j.ccr.2009.11.021 20129247 PMC2818597

[B20] Kennedy SM O'Driscoll L Purcell R Fitz-Simons N McDermott EW Hill AD O'Higgins NJ Parkinson M Linehan R Clynes M Prognostic importance of survivin in breast cancer Br J Cancer 2003 88 1077 1083 10.1038/sj.bjc.6600776 12671708 PMC2376388

[B21] Li YH Hu CF Shao Q Huang MY Hou JH Xie D Zeng YX Shao JY Elevated expressions of survivin and VEGF protein are strong independent predictors of survival in advanced nasopharyngeal carcinoma J Transl Med 2008 6 1 10.1186/1479-5876-6-1 18171482 PMC2254380

[B22] Augello C Caruso L Maggioni M Donadon M Montorsi M Santambrogio R Torzilli G Vaira V Pellegrini C Roncalli M Coggi G Bosari S Inhibitors of apoptosis proteins (IAPs) expression and their prognostic significance in hepatocellular carcinoma BMC Cancer 2009 9 125 10.1186/1471-2407-9-125 19397802 PMC2680906

[B23] Moriai R Tsuji N Moriai M Kobayashi D Watanabe N Survivin plays as a resistant factor against tamoxifen-induced apoptosis in human breast cancer cells Breast Cancer Res Treat 2009 117 261 71 10.1007/s10549-008-0164-5 18815881

[B24] Span PN Tjan-Heijnen VC Manders P van Tienoven D Lehr J Sweep FC High survivin predicts a poor response to endocrine therapy, but a good response to chemotherapy in advanced breast cancer Breast Cancer Res Treat 2006 98 223 230 10.1007/s10549-005-9153-0 16541327

[B25] D'Angelo A Garzia L Andrè A Carotenuto P Aglio V Guardiola O Arrigoni G Cossu A Palmieri G Aravind L Zollo M Prune cAMP phosphodiesterase promotes cancer metastasis by down-regulation of nm23-H1 Cancer Cell 2004 5 137 149 10.1016/S1535-6108(04)00021-2 14998490

[B26] Müller T Stein U Poletti A Garzia L Rothley M Plaumann D Thiele W Bauer M Galasso A Schlag P Pankratz M Zollo M Sleeman JP ASAP1 promotes tumor cell motility and invasiveness, stimulates metastasis formation in vivo, and correlates with poor survival in colorectal cancer patients Oncogene 2010 29 2393 403 10.1038/onc.2010.6 20154719

[B27] Marino N Zollo M Understanding h-prune biology in the fight against cancer Clin Exp Metastasis 2007 24 637 45 10.1007/s10585-007-9109-3 17952613

[B28] Garzia L D'Angelo A Amoresano A Knauer SK Cirulli C Campanella C Stauber RH Steegborn C Iolascon A Zollo M Phosphorylation of nm23-H1 by CKI induces its complex formation with h-prune and promotes cell motility Oncogene 2008 27 1853 64 10.1038/sj.onc.1210822 17906697

[B29] Galasso A Zollo M The Nm23-H1-h-Prune complex in cellular physiology: a 'tip of the iceberg' protein network perspective Mol Cell Biochem 2009 329 149 59 10.1007/s11010-009-0115-4 19390954

[B30] Zollo M Andrè A Cossu A Sini MC D'Angelo A Marino N Budroni M Tanda F Arrigoni G Palmieri G Overexpression of h-prune in breast cancer is correlated with advanced disease status Clin Cancer Res 2005 11 199 205 15671547

[B31] Horii R Akiyama F Ito Y Iwase T Assessment of hormone receptor status in breast cancer Pathol Int 2007 57 784 790 10.1111/j.1440-1827.2007.02174.x 17988279

[B32] Hayward JL Carbone PP Heusen JC Kumaoka S Segaloff A Rubens RD Assessment of response to therapy in advanced breast cancer Br J Cancer 1977 35 292 298 856236 10.1038/bjc.1977.42 PMC2025288

[B33] Sataloff DM Mason BA Prestipino AJ Seinige UL Lieber CP Baloch Z Pathologic response to induction chemotherapy in locally advanced carcinoma of the breast: a determinant of outcome J Am Coll Surg 1995 180 297 306 7874340

[B34] Siddiqa A Long LM Li L Marciniak RA Kazhdan I Expression of HER-2 in MCF-7 breast cancer cells modulates anti-apoptotic proteins Survivin and Bcl-2 via the extracellular signal-related kinase (ERK) and phosphoinositide-3 kinase (PI3K) signalling pathways BMC Cancer 2008 8 129 10.1186/1471-2407-8-129 18454859 PMC2386479

[B35] Lønning PE Breast cancer prognostication and prediction: are we making progress? Ann Oncol 2007 18 Suppl 8 viii3 7 10.1093/annonc/mdm260 17890212

[B36] Rydén L Landberg G Stål O Nordenskjöld B Fernö M Bendahl PO HER2 status in hormone receptor positive premenopausal primary breast cancer adds prognostic, but not tamoxifen treatment predictive, information Breast Cancer Res Treat 2008 109 351 357 10.1007/s10549-007-9660-2 17636399

[B37] Jalava P Kuopio T Juntti-Patinen L Kotkansalo T Kronqvist P Collan Y Ki67 immunohistochemistry: a valuable marker in prognostication but with a risk of misclassification: proliferation subgroups formed based on Ki67 immunoreactivity and standardized mitotic index Histopathology 2006 48 674 682 10.1111/j.1365-2559.2006.02402.x 16681683

[B38] Yerushalmi R Woods R Ravdin PM Hayes MM Gelmon KA Ki67 in breast cancer: prognostic and predictive potential Lancet Oncol 2010 11 174 183 10.1016/S1470-2045(09)70262-1 20152769

[B39] Petit T Wilt M Velten M Millon R Rodier JF Borel C Mors R Haegelé P Eber M Ghnassia JP Comparative value of tumour grade, hormonal receptors, Ki-67, HER-2 and topoisomerase II alpha status as predictive markers in breast cancer patients treated with neoadjuvant anthracycline-based chemotherapy Eur J Cancer 2004 40 205 211 10.1016/S0959-8049(03)00675-0 14728934

[B40] Rouzier R Perou CM Symmans WF Ibrahim N Cristofanilli M Anderson K Hess KR Stec J Ayers M Wagner P Morandi P Fan C Rabiul I Ross JS Hortobagyi GN Pusztai L Breast cancer molecular subtypes respond differently to preoperative chemotherapy Clin Cancer Res 2005 11 5678 5685 10.1158/1078-0432.CCR-04-2421 16115903

[B41] Colleoni M Orvieto E Nolé F Orlando L Minchella I Viale G Peruzzotti G Robertson C Noberasco C Galimberti V Sacchini V Veronesi P Zurrida S Orecchia R Goldhirsch A Prediction of response to primary chemotherapy for operable breast cancer Eur J Cancer 1999 35 574 579 10.1016/S0959-8049(99)00005-2 10492630

[B42] Forus A D'Angelo A Henriksen J Merla G Maelandsmo GM Flørenes VA Olivieri S Bjerkehagen B Meza-Zepeda LA del Vecchio Blanco F Müller C Sanvito F Kononen J Nesland JM Fodstad Ø Reymond A Kallioniemi OP Arrigoni G Ballabio A Myklebost O Zollo M Amplification and overexpression of PRUNE in human sarcomas and breast carcinomas - a possible mechanism for altering the nm23-H1 activity Oncogene 2001 20 6881 6890 10.1038/sj.onc.1204874 11687967

